# How do the sleep features that characterise depression impact memory?

**DOI:** 10.1042/ETLS20230100

**Published:** 2023-12-06

**Authors:** Marcus O. Harrington, Sarah Reeve, Joanne L. Bower, Louis Renoult

**Affiliations:** 1 School of Psychology, University of East Anglia, Norwich, U.K.; 2 Department of Clinical Psychology and Psychological Therapies, Norwich Medical School, University of East Anglia, Norwich, U.K.

**Keywords:** categorical memory, cognitive bias, major depressive disorder (MDD), memory control, REM sleep dysregulation

## Abstract

Depression is associated with general sleep disturbance and abnormalities in sleep physiology. For example, compared with control subjects, depressed patients exhibit lower sleep efficiency, longer rapid eye movement (REM) sleep duration, and diminished slow-wave activity during non-REM sleep. A separate literature indicates that depression is also associated with many distinguishing memory characteristics, including emotional memory bias, overgeneral autobiographical memory, and impaired memory suppression. The sleep and memory features that hallmark depression may both contribute to the onset and maintenance of the disorder. Despite our rapidly growing understanding of the intimate relationship between sleep and memory, our comprehension of how sleep and memory interact in the aetiology of depression remains poor. In this narrative review, we consider how the sleep signatures of depression could contribute to the accompanying memory characteristics.

## Introduction

Sleep and depression have a complex, bi-directional relationship. Impaired sleep can increase vulnerability to depression, whilst depressive symptoms can disrupt sleep and consequently hinder recovery [1–4]. Despite significant recent advances in sleep and psychiatry research, the mechanisms underlying the reciprocal relationship between sleep and depression remain poorly understood. Elucidating these mechanisms could improve the way we treat *major depressive disorder (MDD)*, which affects an estimated 280 million people worldwide [5] and constitutes a major global health challenge.

Memory affords people the ability to relive experiences in rich detail, plan their future, and understand the world they live in. Depression is associated with distinct profiles of memory dysfunction, which increase the risk of developing depression, help to maintain depressive episodes once they have begun, and confer vulnerability to new depressive episodes following remission [6]. Correcting memory dysfunction remains an important aspect of cognitive behavioural therapy (CBT) — the current first-line treatment for depression.

In recent years, scientific understanding of the importance of sleep for memory function has grown exponentially. Despite this growth, the literatures describing sleep and memory dysfunction in depression have remained largely separate. In this review, we evaluate how the sleep features that characterise depression could contribute to memory dysfunctions, including overgeneral autobiographical memory (OGM), emotional memory bias, and impaired memory suppression. For clarity, unfamiliar terms not defined in the main text are italicised on the first usage and defined in a glossary — see Box 1.

**Box 1. Glossary of terms**

*Categorical memories*: memories of events that happened repeatedly over time, in contrast with memories of specific events.

*Declarative memories*: long-term memories that support the conscious recollection of particular facts and events.

*Emotional trade-off task:* a memory task where participants view negative and neutral objects superimposed on neutral scenes, and later have their memory tested for the objects and scenes separately.

*Executive function*: a set of cognitive processes that enable goal-directed behaviour, including planning, decision-making, working memory, cognitive flexibility, and inhibition of irrelevant information or responses.

*Hypersomnia*: difficulty waking up from sleep or staying awake throughout the day.

*Insomnia*: difficulty falling and/or staying asleep, unwanted early morning awakenings, and/or other difficulties in obtaining good quality sleep.

*Major depressive disorder (MDD)*: a psychiatric disorder characterised by persistent low mood, loss of interest or pleasure in activities, and a range of other cognitive, emotional, and physical symptoms.

*Neural oscillations*: the rhythmic patterns of electrical activity that occur in the brain.

*Polysomnography*: the gold-standard tool for measuring sleep objectively, involving the simultaneous recording of brain activity, muscle tone, and eye movements.

*Procedural memories*: long-term memories involved in the acquisition and retention of skills or habits, that are typically expressed through performance rather than explicit knowledge (e.g. riding a bike, typing, or playing an instrument).

*REM density*: the frequency of rapid eye movements per unit of REM sleep.

*REM sleep latency*: the length of time between the onset of sleep and the onset of REM sleep.

*Sleep efficiency*: the proportion of time spent asleep relative to the total time dedicated to sleep.

*Sleep latency*: the length of time between initially attempting to go to sleep and the onset of actual sleep.

## What sleep features characterise depression?

### General sleep disturbance

Depression is associated with sleep issues, particularly *insomnia* and *hypersomnia*. In a recent population-based study, 92% of participants with MDD reported some kind of sleep disturbance, with 48.5% reporting insomnia, 13.7% reporting hypersomnia, and 30.2% reporting co-occurring insomnia and hypersomnia [7]. Given this overlap, it is unsurprising that sleep issues have traditionally been considered secondary diagnostic criteria for depression. This framing neglected the possible role of sleep as a causal factor in depression. However, more recently, studies have demonstrated that insomnia increases the risk of depression onset and relapse [8,9], is associated with more severe symptoms and worsened functioning amongst those with depression [10,11], often does not remit during treatment for depression [12], and inhibits response to depression treatment [13]. Consequently, clinical guidance has shifted to advise that where sleep disorder symptoms meet clinical thresholds, they should be considered as a comorbid disorder and receive appropriate treatment [14]. This new perspective of considering insomnia as a treatable comorbidity has led to the discovery that CBT for insomnia (CBTi), as either a standalone or integrated component of depression treatment, improves sleep and mood outcomes [15–18], and can prevent depression incidence or relapse [19].

Diagnosis of insomnia is reliant on self-reported accounts of sleep disturbance or reduced sleep quality [14]. Importantly, however, *polysomnography* studies reveal that depressed patients (vs non-clinical controls) also exhibit objective evidence of poor sleep, including lower *sleep efficiency* [20–24], longer *sleep latency* [20–22,24], and shorter total sleep time [20,21,23,24].

### Abnormalities in sleep physiology

**Figure 1.**
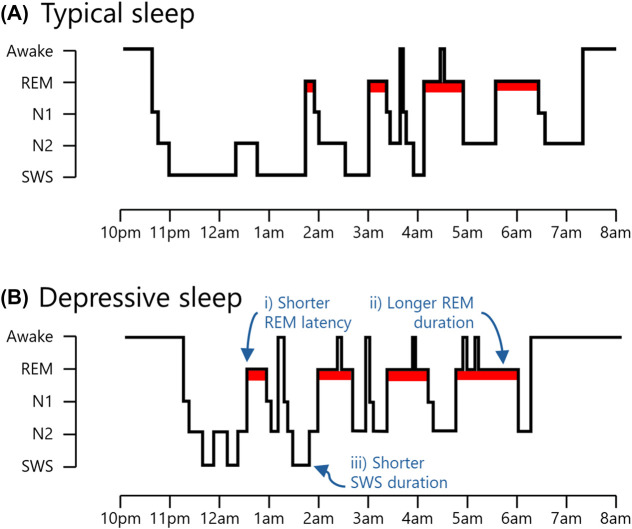
Depiction of typical sleep and sleep in depression. (**A**) Sleep can be broadly classified into two main types: rapid eye movement (REM) sleep, and non-rapid eye movement (NREM) sleep. The latter can be further divided into three stages, with each stage signifying progressively deeper levels of sleep. The deepest stage of NREM sleep is often referred to as slow-wave sleep (SWS) because it is defined by the presence of low-frequency *neural oscillations* (<4 Hz). In contrast, REM sleep is defined by the presence of darting eye movements and is associated with comparatively fast, mixed-frequency oscillations (∼4–25 Hz). Across the night, NREM and REM sleep occur sequentially in ∼90 min cycles. The ratio of SWS to REM sleep shifts throughout the night, with most SWS occurring in the first half of the night, and most REM sleep occurring in the second half of the night. (**B**) The overall structure of sleep is broadly similar in depressed patients. However, compared with non-depressed individuals, depressed patients exhibit (i) shorter REM sleep latency (i.e. they enter REM sleep more quickly following sleep onset), (ii) longer REM sleep duration (i.e. they spend more time in REM sleep), and (iii) shorter SWS duration (i.e. they spend less time in SWS). Notably, depressed patients also exhibit greater REM density (i.e. more rapid eye movements during REM sleep) and exhibit less low-frequency (<4 Hz) brain activity during SWS (not depicted). Abbreviations: N1 & N2, light stages of NREM sleep.

Human sleep is not a single state, but rather a sequence of distinct states — known as sleep stages — that unfold in a cyclical pattern across the night. For an overview of sleep stages and structure, see Figure 1A.

It has long been known that several physiological sleep features distinguish participants with depression from their non-depressed counterparts [25–27]. Empirical work in this area has produced mixed findings, in part because of small sample sizes and methodological differences between studies. However, meta-analyses have found depression to be associated with shorter *REM sleep latency* [20,22–24], greater *REM density* [20–24], longer REM sleep duration and/or proportion [20,22–24], and shorter SWS duration and/or proportion [23,24] (Figure 1B). For a summary of meta-analytic findings, see Table 1.

**Table 1:** Summary of meta-analyses investigating sleep polysomnography in depressed patients

Authors (year)	Participants	Greater REM density	Shorter REM sleep latency	Longer REM sleep duration/proportion	Shorter SWS duration/proportion	Longer N1 duration^1^	Shorter N2 duration^1^
Baglioni et al. [20]	Adults: MDD vs control	⧫	⧫	⧫	◊	⧫	⧫
Lovato & Gradisar [21]	Adolescents: MDD vs control	⧫	◊	◊	◊	◊	◊
Augustinavicius et al. [22]	Children/adolescents: MDD vs control	⧫	⧫	⧫	◊	◊	◊
Pillai et al. [23]	Adults: MDD vs control	⧫	⧫	⧫	⧫	○	○
Benca et al. [24]	Adults: affective disorder vs control	⧫	⧫	⧫	⧫	○	○

⧫ = positive result; ◊ = negative result; ○ = not investigated/reported.

1 Abnormalities in the duration of non-rapid eye movement (NREM) sleep stages N1 and N2 are included in this table for completeness but are not discussed in the main text.

Abbreviations: MDD, major depressive disorder; REM, rapid eye movement; SWS, slow-wave sleep.

The *neural oscillations* generated during sleep also differ between depressed and non-depressed participants [28–32]. Although findings in this area have also been inconsistent, several studies have converged on the finding that the low-frequency oscillations (<4 Hz) that hallmark SWS are diminished in depressed participants [33–36], particularly during the first sleep cycle [37,38].

Sleep is highly heritable [39–41], and indeed some of the sleep features that hallmark depression have also been observed in non-depressed first-degree relatives of patients [42–44]. Relatives of depressed patients who exhibit sleep features consistent with depression are at higher risk of developing depression than those who do not exhibit such sleep features [43,45,46]. Moreover, although some depressive sleep features become less prominent during periods of remission [23], their persistence beyond depressive episodes may predict subsequent relapse [47,48]. Findings such as these suggest that, alike general sleep disturbance, abnormalities in sleep physiology often precede the onset of depression, and may be involved in its aetiology.

Before progressing, we must again highlight that many of the findings introduced in this section are borne out of dated studies with small sample sizes. This area of research would benefit from new data obtained from large cohort studies.

## How do these sleep features impact memory?

### Overgeneral autobiographical memory

Autobiographical memory refers to a person's capacity to remember personal facts and events. Within autobiographical memory, people typically differentiate memories of specific personal events (e.g. ‘I got a flat tyre on my drive to work last Saturday’) from more general *categorical memories* (e.g. ‘I drove to work every day throughout the winter’). One of the most widely used tests to evaluate autobiographical memory is the autobiographical memory test (AMT) [49–52], where participants are asked to retrieve a specific personal memory — which is generally defined as context-specific (happening at a specific time and place) and lasting less than a day — in response to a cue word (e.g. ‘car’).

Patients with depression consistently produce less specific personal events and more general categorical memories in the AMT [53,54]. This phenomenon — known as overgeneral autobiographical memory (OGM) — is associated with poorer prognosis and more severe depressive symptoms in follow-up clinical evaluations [55,56]. Moreover, interventions aimed at enhancing the specificity of autobiographical memory recall have proven effective in reducing depressive symptoms [57]. Findings such as these suggest that OGM may contribute to the onset and maintenance of depression [58].

One important mechanism underlying OGM in depression is impaired *executive function* [59]. Briefly, when people search for specific personal events related to a cue, as in the AMT, their search is thought to typically begin at the categorical level (e.g. driving to work in the winter) which activates the retrieval process for specific personal events within that category (e.g. getting a flat tyre on the way to work last Saturday), in addition to other interfering information [60]. Due to impaired executive function, depressed individuals truncate their search at the categorical level, in part because they do not have the capacity to inhibit interfering information while a suitable specific memory is located [54,59].

Executive function declines under conditions of poor sleep. Indeed, after acute sleep deprivation (vs sleep), participants exhibit poorer performance on a variety of executive function tasks, including tasks that require the inhibition of unwanted or interfering information [61–65]. Moreover, in a recent, large-scale study (*n* = 479 420), executive function was found to be sensitive to individual differences in self-reported habitual sleep duration [66]. Executive function was greatest in individuals who reported sleeping for 7 h per night. In individuals who reported sleeping less than 7 h per night, increasingly shorter sleep duration was associated with increasingly poorer executive function. Finally, a systematic review of the link between sleep and executive function in older adults suggests that other sleep parameters besides short sleep duration, including low sleep efficiency, are also linked to executive function impairments [67].

Recent work has provided direct evidence that poor sleep is associated with OGM. For example, a tendency to recall fewer specific autobiographical memories in the AMT has been observed following acute sleep deprivation [68] and is also associated with shorter habitual sleep duration [69]. Moreover, individuals with *obstructive sleep apnea (OSA) —* a sleep disorder associated with repeated episodes of interrupted breathing and, consequently, disrupted sleep — recall fewer specific autobiographical memories in the AMT than participants without OSA [70,71].

Few studies that have examined the relationship between sleep and autobiographical memory looked at the role of specific sleep stages. However, there is some evidence that SWS, which is impoverished in depression, may be important for the formation of autobiographical memories. For example, in a study of individuals with early-stage Alzheimer's disease, longer overnight SWS duration was associated with superior next-day recall of recently acquired autobiographical memories [72]. Moreover, in trauma-exposed females, greater overnight SWS proportion was associated with the production of more specific autobiographical memories in response to emotionally neutral cues (but not positive or negative cues) in an AMT task the following morning [73].

In sum, poor sleep may represent an important mechanism underlying OGM in depression, possibly via its detrimental effect on executive function. Impoverished SWS could generally impair autobiographical memory, although this area of research is still in its infancy.

### Emotional memory bias

The process of learning and remembering involves three stages: encoding (transforming sensory information into a neural code that can be stored in memory), storage/consolidation (maintaining/stabilizing that information over time), and retrieval (accessing and using that stored information when desired). Emotional memory refers to the storage and retrieval of memories that elicited an emotional reaction at the time of encoding.

Emotionally negative memories are typically retained for longer than memories not associated with strong emotions [74–76]. There are clear evolutionary advantages to prioritising negative memories over neutral ones. For example, survival is more likely to benefit from remembering an encounter with a venomous snake than a harmless rabbit. In depression, however, this bias towards negative memories may be unduly accentuated. Indeed, a recent meta-analysis of 154 independent studies demonstrated that emotional memory bias is enhanced in depressive participants, as compared with non-depressive control participants [77]. This effect was also observed in remitted participants (vs never-depressed participants) under conditions of induced stress or negative mood [77], supporting the view that emotional memory bias represents a vulnerability factor for depression [6,78].

Importantly, there is substantial evidence that sleep problems can lead to emotional memory bias by influencing encoding, consolidation, and retrieval processes.

#### Emotional bias in memory encoding

Emotional memory bias can begin at the time of encoding — if somebody pays attention to the negative aspects of an event, they perceive that event more negatively, and ultimately remember the event as being negative [78,79]. Poor sleep can precipitate emotional biases in attention and perception. Indeed, in individuals with elevated negative thinking, self-reported shorter habitual sleep duration and longer sleep onset latency are associated with increased difficulty disengaging attention from negative (vs neutral) images [80]. Moreover, in young adolescents, objectively poorer sleep is associated with deficits in detecting positive facial expressions [81]. Experimental sleep deprivation studies have produced similar findings. As compared with rested participants, sleep-deprived participants are less effective at inhibiting responses to negative (but not positive) words, indicative of heightened attention towards negative information [82]. Furthermore, in the morning after a night of sleep deprivation, participants rate neutral images more negatively than they did the evening prior [83]. This effect was not observed in a control group who slept, suggesting that sleep deprivation causes ambiguous information to be interpreted in a negative light.

In sum, sleep disturbances in depression could set the scene for emotional memory bias by directing attention towards negative information and colouring daytime experiences with increased negative emotionality.

#### Emotional bias in memory consolidation

During sleep, the brain is shielded from new sensory input, creating an environment that is ideally suited to processing and consolidating recently encoded memories [84–86]. Several studies using a range of experimental tasks have shown that negative memories (vs neutral memories) are more likely to be retained over sleep than wakefulness [87–90]. Although the robustness of this effect is a topic of ongoing debate [91,92], the effect has been observed relatively consistently in studies that used the *emotional trade-off task*. Such studies typically show that sleep selectively enhances memory for emotional objects at the expense of neutral background details [89,93–97].

REM sleep may be particularly important for the consolidation of emotionally salient memories [88,94,98–101]. For example, greater retention of emotional *declarative memories* across sleep episodes is associated with longer REM sleep duration and/or proportion [88,94,100], shorter REM sleep latency [88], and greater REM density [98,99]. Accordingly, it can be hypothesised that REM sleep dysregulation may increase vulnerability to depression by disproportionately amplifying the strength of aversive memories [102–104]. At present, this notion lacks direct empirical support. However, small studies have found some evidence that in individuals reporting depressive symptoms, REM sleep may be particularly amenable to emotional memory retention [98], and conversely, sleep deprivation may be particularly devastating for emotional memory retention [105].

Whereas REM sleep prioritises the retention of emotional memories, SWS is thought to be important for the retention of neutral memories [84–86]. Indeed, the slow oscillations that hallmark SWS are believed to play a causal role in consolidating neutral declarative and *procedural memories* [106].

Depressed participants exhibit deficient retention of neutral memories [107]. This deficit could exacerbate emotional memory bias by further increasing the ratio of negative to neutral memories and may be attributable to impoverished SWS. Offering some support to this view, non-depressed participants exhibit sizeable improvements in a procedural finger-tapping task after a night of sleep, but depressed participants exhibit smaller improvements [108,109], or even a decline in performance [110,111]. The sleep-associated consolidation of neutral declarative memories in depression has received less empirical attention. However, one study has indicated that although depressed participants exhibit poorer initial encoding of neutral declarative memories than non-depressed participants, their overnight retention is largely unaffected [111].

In sum, the features of REM and NREM sleep that characterise depression may both contribute to the development of emotional memory bias. Specifically, enhanced REM sleep could disproportionately bolster aversive memories, whereas impoverished SWS could impair neutral memory retention, exacerbating the predominance of aversive memories.

#### Emotional bias in memory retrieval

The memory retrieval process is also susceptible to emotional bias — under certain conditions, negative memories may be more accessible than their neutral counterparts. To our knowledge, no studies have specifically and directly examined the impact of sleep on the retrieval of negative vs positive or neutral information. However, it is well-established that impaired sleep is associated with increased negative affect [112,113], which reliably induces emotional biases in memory retrieval (and encoding) [114] — a phenomenon known as mood-congruent memory. For example, in non-clinical participants, induction of a sad mood leads to superior recall of previously learned negative words (vs positive words), whereas induction of a happy mood leads to the opposite pattern of results [115]. Moreover, when asked to recall personal events from their high-school years, non-clinical participants in an induced sad mood are more likely to recall negative events, as compared with those in a neutral mood [116]. These results indicate that negative affect — a robust consequence of poor sleep — improves the accessibility of negative memories.

Negative memories may be more accessible after waking from REM sleep as compared with NREM sleep. In one study, participants completed an AMT shortly after being awoken from either REM or NREM sleep [117]. Across the sample, 49% of autobiographical memories produced in response to a neutral cue word were negative after REM sleep awakenings, compared with just 30% after NREM sleep awakenings, indicating that REM sleep may be conducive to biased emotional memory retrieval. In participants with higher self-reported symptoms of depression/anxiety, this proportion increased to 67% and 35% for awakenings from REM and NREM sleep, respectively.

In sum, sleep disturbances associated with depression could indirectly facilitate emotional biases in memory retrieval by promoting negative affect. Moreover, REM sleep dysregulation could render negative memories hyper-accessible during subsequent wakefulness, although this notion requires further investigation. More generally, additional research is required to better understand the impact of sleep on the encoding, consolidation, and retrieval of emotional memories. For an excellent summary of outstanding issues and concrete suggestions for future research, please see ref. [91].

### Deficient memory suppression

In everyday life, individuals can encounter reminders of unpleasant past experiences, which can trigger the retrieval of unwanted memories. For example, the sight of a red car driving too quickly may cause somebody to bring to mind a past motor vehicle accident involving a red car. When unwanted memories intrude, people often directly suppress the retrieval of those memories, thereby clearing their consciousness of unwelcome reminders of the past. Memory suppression can be measured in the laboratory using the think/no-think (TNT) task [118], where participants learn associations between pairs of items (e.g. word pairs or face-image pairs) and then repeatedly attempt to either retrieve or suppress one item when cued with the other. Studies using the TNT task have consistently demonstrated that repeatedly suppressing an item leads to impaired recall of that item during later memory tests [119–121], a phenomenon known as suppression-induced forgetting (SIF).

Importantly, SIF is less robust in depressive individuals than in control participants [122], indicative of deficient memory suppression. This inability to effectively suppress unwanted memories could have far-reaching consequences for the aetiology of depression. For example, successful memory suppression diminishes the emotional intensity of unpleasant past experiences, rendering those memories less aversive upon subsequent recollection [63,123]. Accordingly, considered alongside the SIF literature, deficient memory suppression could cause both the mnemonic content and emotional tone of unpleasant memories to endure, which could impede efforts to overcome trauma. Moreover, suppressing the retrieval of a specific memory reduces the likelihood of that memory intruding into consciousness in the future [63,123–125], thereby curbing recurring memory intrusions. Deficits in memory suppression could therefore cause or exacerbate intrusive thoughts, repetitive thinking, and rumination, which are all prevalent in depression [126–129].

As previously discussed, sleep loss can have a devastating impact on a variety of executive functions, including memory suppression. Indeed, in a recent study, sleep-deprived participants were poorer than rested participants at keeping emotionally negative and neutral scenes out of mind when presented with reminder cues in a TNT task [63]. Consistent with the view that deficient memory suppression can have lasting consequences, the benefit of memory suppression in reducing future memory intrusions was less robust in sleep-deprived participants (vs rested participants). Moreover, whereas memory suppression led to reductions in subjective and psychophysiological emotional responses to negative scenes in rested participants, these effects were not observed in sleep-deprived participants.

In addition to impairing the ability to keep unwanted memories at bay when faced with reminder cues, poor sleep may also precipitate spontaneous intrusive memories about traumatic experiences. Indeed, prolonged wakefulness (vs sleep) in the aftermath of a laboratory-based trauma (e.g. watching a disturbing film clip) causes intrusive memories about the traumatic event to be more frequent [130–132] and subjectively distressing [131]. Moreover, greater subjective sleep disturbance in the first week after experiencing or witnessing a real-life motor vehicle accident is associated with more frequent memory intrusions [133]. Conversely, taking a short nap after experiencing laboratory-based trauma curbs subsequent memory intrusions [134]. It should be noted that results in this domain have not been entirely uniform across the board. In a pair of studies by the same research group, typical sleep (vs acute sleep deprivation) was associated with impaired memory suppression in the days immediately following exposure to distressing film clips [135,136]. Critically, however, after this initial deficit, the effect either dissipated [135] or reversed [136], indicating that any potential memory suppression deficits after sleep were short-lived.

The notion that memory suppression can be both effective and beneficial is controversial. For opposing views, see refs [137] and [138]. This controversy is fuelled partly by the literature on thought suppression as explored through the ‘white bear task’ [139]. In those studies, when participants are instructed to avoid thinking about (i.e. suppress) a specific item (e.g. a white bear) for 5 min, they typically think about the item more often than they otherwise would have done both during and after the 5 min suppression period. Importantly, although this task is conceptually similar to the TNT task typically used in the memory suppression literature, it has been argued that the white bear task fails to capture the mechanistic deficits that lead to real-life memory intrusions [140], an argument supported by evidence that performance in the white bear task is not impaired in clinical samples [141].

Clinical concerns about memory suppression also stem from the conception it is a maladaptive avoidance behaviour that ultimately perpetuates mental health issues [137]. It is difficult to ascertain whether such concerns are wholly unfounded. It should be noted, however, that suppressing memories in the TNT task involves repeatedly confronting reminders to unwanted memories, rather than avoiding them entirely. Moreover, recent work has demonstrated that training people to suppress feared events does not exacerbate psychiatric symptoms [138]. Conversely, training led to a reduction in subjective distress for feared events and a sustained alleviation of depressive symptoms.

In sum, memory suppression may play an essential role in mental well-being, and its deficiency may be implicated in the aetiology of depression. Recent research has identified sleep loss as an important factor underlying failures of memory suppression, leading to the hypothesis that impaired memory suppression may bridge the gap between sleep disturbance and increased vulnerability to psychiatric disorders [142].

## Conclusion

**Figure 2.**
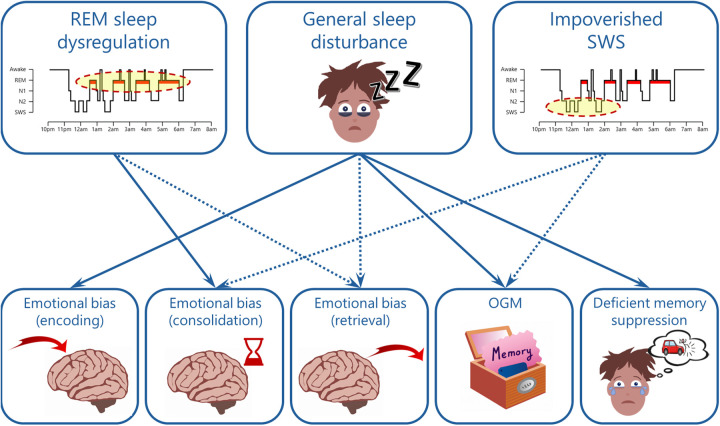
An overview of possible links between the sleep and memory features that characterise depression. Solid arrows reflect links that are reasonably well-established by experimental data, whereas dotted arrows reflect links that are indirect or less established. Additional links are possible and may emerge through further empirical investigation. Moreover, bi-directional links are likely (e.g. deficient memory suppression could lead to general sleep disturbance), but beyond the scope of the present article. Abbreviations: REM, rapid eye movement; SWS, slow-wave sleep; N1 & N2, light stages of NREM sleep; OGM, overgeneral autobiographical memory.

Distinct patterns of dysfunction in sleep and memory have been independently identified as important factors underlying the onset and maintenance of depressive episodes. Considering that sleep is centrally involved in a variety of memory functions, it seems likely that the sleep features that characterise depression give rise to the patterns of memory dysfunction associated with the disorder (Figure. 2). In support of this view, the literature reviewed in this article demonstrates that after sleep loss, non-clinical participants often exhibit profiles of memory disturbance consistent with depression. The literature also highlights memory dysfunction as a potential mechanism underlying the role of physiological sleep abnormalities (e.g. REM sleep dysregulation and impoverished SWS) in depression aetiology.

It is pertinent to acknowledge that MDD frequently co-occurs with other psychiatric disorders, with anxiety disorders being among the most common comorbid conditions [143,144]. Importantly, alike many other psychiatric illnesses, anxiety disorders have been linked to reduced sleep quality [145] and polysomnographic sleep abnormalities [146,147]. Moreover, individuals with anxiety disorders also exhibit depression-related memory dysfunctions, including accentuated emotional memory bias [148] and impaired memory suppression [122]. Such findings demonstrate that the sleep and memory abnormalities discussed in this article are not exclusive to depression. They also highlight the need for further research aimed at disentangling the aetiology of depression from that of other psychiatric disorders.

A second caveat to consider is that many studies supporting the idea that sleep loss contributes to depression-related memory dysfunction used acute sleep deprivation designs. Although such studies can shed light on the implications of clinical sleep disruptions, they may not accurately represent the broad spectrum of sleep disturbances associated with depression. Indeed, in a typical study designed to assess the impact of sleep deprivation on memory, participants undergo a single night of total sleep deprivation, and their next-day memory performance is compared with a control group that slept normally. This is qualitatively different from clinical sleep disruption, which may be better captured using prolonged partial sleep restriction protocols (e.g. [112]).

An important challenge for future research will be to elucidate the extent to which memory dysfunction mediates the link between impaired sleep and depression. Such efforts would be facilitated by the routine inclusion of depressive symptomatology measures in sleep and memory studies and, likewise, the inclusion of sleep measures in cognitive studies of participants with depression. A closer alignment of these two exciting fields of research would help to shed light on the complex interplay between sleep, memory, and depression, facilitating treatment strategies for depressed patients and preventative measures designed to ward off depression in poor sleepers.

## Summary

- Depression is associated with sleep disturbance and abnormalities in sleep physiology. Most notably, rapid eye movement (REM) sleep is more abundant, whereas slow-wave sleep (SWS) is reduced.
- Depression is also associated with distinct patterns of memory dysfunction, including overgeneral autobiographical memory (OGM), emotional memory bias, and impaired memory suppression.
- Sleep and memory characteristics of depression may be interrelated, whereby depressive sleep features lead to memory dysfunction and ultimately increase vulnerability to depression onset, maintenance, or relapse.
- Poor sleep could underlie OGM, emotional biases in memory encoding and retrieval, and impaired memory suppression.
- Elevated REM sleep could enhance emotional memory consolidation and retrieval, whereas reduced SWS could exacerbate emotional memory bias and impair autobiographical memory.

## Open Access

Open access for this article was enabled by the participation of University of East Anglia in an all-inclusive *Read & Publish* agreement with Portland Press and the Biochemical Society under a transformative agreement with JISC.

## Abbreviations

AMT: autobiographical memory test
CBT: cognitive behavioural therapy
MDD: major depressive disorder
NREM: non-rapid eye movement
OGM: overgeneral autobiographical memory
OSA: obstructive sleep apnea
REM: rapid eye movement
SIF: suppression-induced forgetting
SWS: slow-wave sleep
TNT: think/no-think
